# Age at menarche and the future risk of gestational diabetes: a systematic review and dose response meta-analysis

**DOI:** 10.1007/s00592-018-1214-z

**Published:** 2018-08-29

**Authors:** Clive J. Petry, Ken K. Ong, David B. Dunger

**Affiliations:** 1 Department of Paediatrics, University of Cambridge, Box 116, Cambridge Biomedical Campus, Hills Road, Cambridge, CB2 0QQ UK; 2 Medical Research Council Epidemiology Unit, University of Cambridge, Cambridge, CB2 0QQ UK; 3 The Institute of Metabolic Science, University of Cambridge, Cambridge, CB2 0QQ UK

**Keywords:** Menstruation, Puberty, Pregnancy, Random effects

## Abstract

Published studies show an inconsistent association between age at menarche and the subsequent risk of developing gestational diabetes mellitus when pregnant. This systematic review and meta-analysis was performed to clarify any trends in this association in published observational population studies. We searched online databases for relevant studies, entered into them up until June 21st 2017. Five eligible studies were found and a pooled random effects dose response meta-analysis of results from these was conducted. This included coverage of 58,133 pregnancies, from which 3,035 women developed gestational diabetes. There was evidence of a non-linear association between age at menarche and gestational diabetes (overall *p* = 1.4 × 10^−8^; *p* for non-linearity = 2.4 × 10^−4^), along with evidence of relatively low heterogeneity (*I*^2^ = 25.5%). The largest predicted risk of gestational diabetes was associated with having a low age at menarche; the mean (95% confidence interval) risk relative to that associated with menarche at age 13 years being: 9 years 2.0 (1.6, 2.4), 10 years 1.6 (1.4, 1.9), 11 years 1.3 (1.2, 1.4), 12 years 1.1 (1.1, 1.1), 13 years was the reference, 14 years 1.0 (1.0, 1.0), 15 years 1.1 (0.9, 1.2), 16 years 1.1 (0.9, 1.4). There was evidence of potential publication bias, such that the maximal true relative risk of gestational diabetes, associated with an age at menarche of 9 years, may be closer to 1.6 than 2. Nevertheless, the curvilinear relationship between age at menarche and the future risk of gestational diabetes in pregnancy appears robust.

**Electronic supplementary material:**

The online version of this article (10.1007/s00592-018-1214-z) contains supplementary material, which is available to authorized users.

## Introduction

The relationship between age at menarche (AAM) and the subsequent risk of developing gestational diabetes (GDM) in pregnancy is a potentially important one for preventive medicine as there has been a generalised global lowering of AAM in the last century [1] combined with an increasing prevalence of GDM [2]. This increasing prevalence of GDM is thought to be one of the factors fuelling the current and future predicted worldwide diabetes epidemic [3]. The strong links between GDM development in pregnancy and the future development of type 2 diabetes (women who experienced GDM having a greater than seven times higher risk of developing type 2 diabetes than those with normoglycaemic pregnancies [4]), and the fact that in utero exposure to GDM increases the number of GDM risk factors female babies have when they get pregnant as adults [5, 6], means that being able to predict those women most at risk of GDM when they become pregnant may be important in targeting lifestyle interventions. Treating susceptible women earlier may become possible [7], which could help reduce the incidence of associated complications.

Partially due to shared genetic risk factors [8], it has been suggested that GDM represents an early manifestation of type 2 diabetes [9], with the physiological insulin resistance of pregnancy causing the premature expression of the disease. Risk of type 2 diabetes has already been shown to be associated with AAM [10], including by use of a Mendelian randomisation approach [11]. As long ago as 1975 it was suggested that AAM may be linked to the development of GDM [12], although it was not formally tested. More recently, various studies have investigated links between AAM and the risk of developing GDM in pregnancy and whilst some significant associations have been found [13–17] (with maximal relative risks for GDM, associated with the earliest AAMs relative to those at the median, ranging from 1.3 to 3.7), this is not true of all studies [18]. Even where a relationship has been observed, sometimes the significantly higher relative risk of GDM is just associated with earliest AAM [13] whereas in other studies a more curvilinear relationship between AAM and GDM is evident (even if it were not always formally tested for or statistical significance reached [14–18]), albeit smaller in magnitude than that associated with the earliest AAMs. This systematic review and meta-analysis was therefore designed to clarify the relationship between AAM and the risk of developing GDM in pregnancy, both in terms of its association and the shape of that association.

## Materials and methods

The present systematic review was performed in accordance with the Preferred Reporting Items for Systematic Reviews and Meta-Analyses (PRISMA) [19] (Online Resource 1). No prior review protocol exists for this analysis.

### Search strategy

On 21st June 2017, we searched relevant online databases with the search strings “age at menarche AND gestational diabetes” and “‘age at menarche’ AND ‘gestational diabetes’”. Although all the databases were presented in English, no account was taken of the language that the study was published in. Neither was any date range of publication adhered to. The online databases and the number of papers/abstracts highlighted by that database were (with the main papers duplicated in all the databases): Pubmed (http://www.pubmed.gov; contains more than 28 million citations from MEDLINE, life science journals and online books; 24 papers highlighted), Web of Science (http://www.webofknowledge.com; contains more than 151 million records from academic journals, books and proceedings, plus patents and data sets; 14 papers highlighted), Scopus (http://www.elsevier.com/solutions/scopus; contains more than 1.4 billion cited references from peer-reviewed journals, conference papers, books, trade publications and patents; 20 papers highlighted) and Turning Research Into Practice (http://www.tripdatabase.com; a clinical search engine designed for searching for high-quality evidence to support clinical practice and care: searches in key medical journals, Cochrane Systematic reviews, clinical guidelines, and other relevant websites; 67 papers highlighted). Over the following months, the abstracts, summaries and/or full papers were then screened and considered relevant if they related directly to an association between AAM and future GDM risk, of which there were a total of 11. Whilst the majority of this searching and screening procedure was performed by one investigator (CJP), consensus agreement was reached between all three investigators (all of whom were qualified to at least PhD and/or MD standard) as to what should be included in the final meta-analysis. We knew of no studies other than our own that had been completed in this subject area but which had not been published.

### Eligibility

**Fig. 1:**
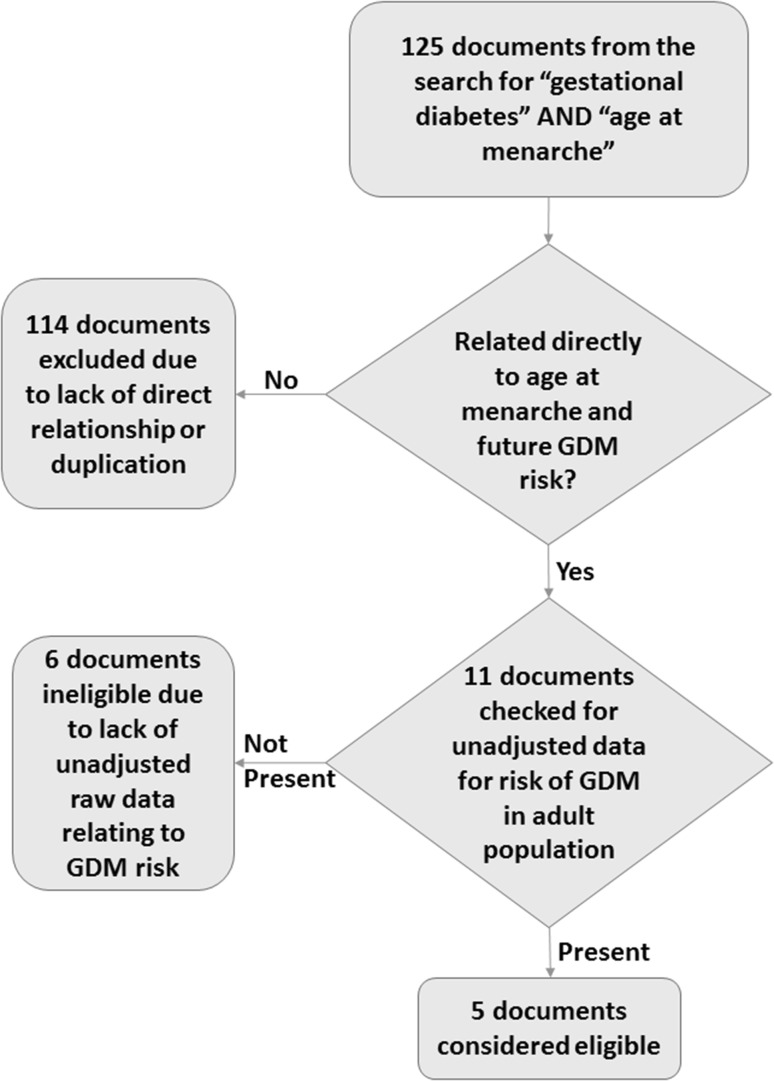
A flow diagram illustrating the systematic process of choosing and eliminating studies for the meta-analysis relating age at menarche with the future risk of GDM in pregnancy

With the exception of our own study [17] which was not published by this date but which we were obviously aware of, documents were considered eligible if they described observational studies of adult populations (cohort, case/control, cross-sectional or any combinations of these) of women and were published in at least an abstract form from which data could be extracted. They also needed to present data that either included crude (unadjusted) numbers of women categorised according to whether or not they had GDM in their pregnancies according to AAM or odds ratios/relative risks for GDM. The total number of studies considered eligible for meta-analysis, including our own, was five all of which were considered high quality (five studies at this stage were rejected since they did directly relate AAM with risk of GDM). All of the studies that were eligible for the meta-analysis were written and published in English. The reference lists for each of these manuscripts were checked manually for any potentially suitable additional studies but failed to include any that were not already accounted for. In one additional study that was excluded [16], relative risks were presented but only data already adjusted for age and ethnicity were included in the manuscript. The number of women with and without GDM in each category of AAM was not calculable, so this manuscript was not included in the meta-analysis. Figure 1 presents a flow diagram of the different stages of searching and screening process for eligible studies.

### Statistical analysis

Unless they were already presented in the manuscript [15], relative risks were calculated from raw crude data where it was available [13, 17, 18], with the AAM category that encapsulated 13 years as the reference. If the risk data was presented just as odds ratios [14] and the prevalence of GDM in the age 13 at menarche category was not calculable, the overall population prevalence was calculated and used as a best estimate instead. Relative risks were then calculated using the method of Zhang and Yu [20]. We therefore finished with a number of relative risks in different AAM categories (a dose–response curve). Meta-analysis of the dose response curves was then performed using the R package dosresmeta [21–23] to indicate different risks of GDM at each age of AAM relative to that at age 13.

Sensitivity analysis was performed in two different ways. For the first one, we repeated the dose response meta-analysis adding one further relevant study that had been excluded from the main meta-analysis because the group sizes had to be estimated, unlike in the other studies, only first pregnancies were studied and the only relative risks for GDM that were presented were already adjusted for age and ethnicity [16]. Including this study in the meta-analysis increased the number of pregnant study participants to 64,047, including 3,236 women that developed GDM. The second form of sensitivity analysis was performed by excluding each of the 5 original studies in the main meta-analysis one at a time in turn to see if the associations in the pooled random effects dose response meta-analysis remained significant without that study being included.

Publication bias at the outcome level was assessed using Egger’s regression test [24, 25] and funnel plots using the R package metafor [26]. As Egger’s test was not designed to be used with a dose response curve, we modified it such that each of our studies was entered twice into the test (pooling the age-related categories below that containing age 13 as one entry, and those categories above that containing age 13 as the other entry, using those categories containing age 13 as the reference). To assess potential effects of publication bias on relative risks, the meta-analysis was performed just including the largest study [13], then just the largest and second largest studies [13, 14] and then adding one study at a time in decreasing overall size order until all five studies were included in the meta-analysis, as suggested by Borenstein et al. [27].

Statistical analysis was performed using R (version 3.4.1; R Foundation for Statistical Computing, Vienna, Austria) or Stata (version 13.1; StataCorp LP, College Station, TX, USA). *p* < 0.05 was considered statistically significant throughout. Data are presented as mean (95% confidence interval) unless otherwise stated.

### Data availability

Data sharing is not applicable to this article as no datasets were generated during the current study.

## Results

### Meta-analysis

**Fig. 2:**
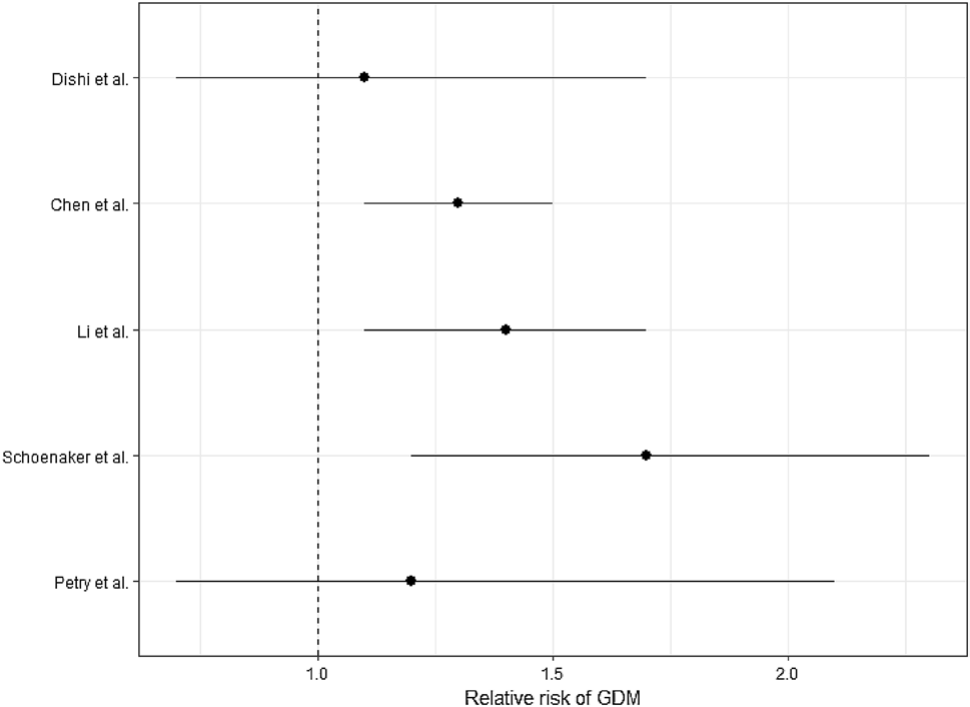
Forest plot of the relative risks for GDM in women with an AAM ≤ 11 years relative to that of women with an AAM of 13 years in citations [13–15, 17, 18], respectively

**Fig. 3:**
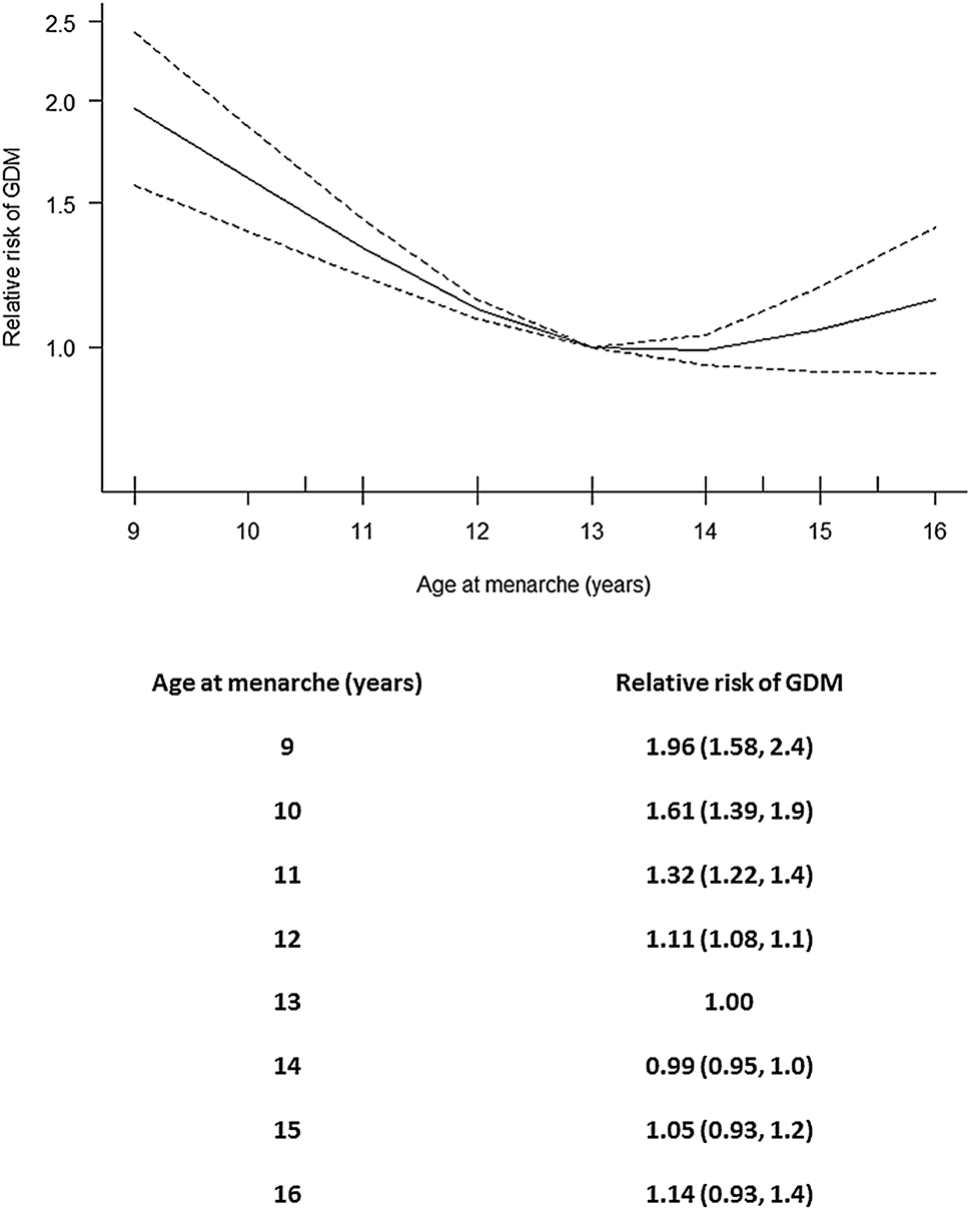
Pooled dose–response association between age at menarche and the risk of developing GDM in pregnancy (represented by the solid line) relative to the risk associated to an age of menarche of 13 years. Age at menarche was modelled with restricted cubic splines in a multivariate random effects dose response model. The relative risks of developing GDM are plotted on the log scale. The dashed lines show the 95% confidence intervals for the spline model

Prior to the meta-analysis inspection of the Forest Plot revealed a possible increased relative risk of GDM in women with an age of menarche ≤ 11 years relative to one of 13 (Fig. 2). The meta-analysis included results from 58,133 pregnancies included in the five different studies (Table 1), of whom 3035 were affected by GDM. We found evidence for a non-linear association between AAM and the risk of developing GDM in pregnancy (overall *p* = 1.4 × 10^−8^; *p* for non-linearity = 2.4 × 10^−4^). The multivariate Cochran Q-test for heterogeneity was not statistically significant: *Q* = 10.7 (8 degrees of freedom), *p* = 0.2, *I*^2^ = 25.5%. Figure 3 shows the meta-analysed predicted relationship between AAM and the risk of developing GDM in pregnancy.

**Table 1:** A summary of the studies that were included in the meta-analysis

Source, country	Average age at menarche (years)	Average age at pregnancy study (years)	Study population	Number of pregnancies affected by GDM and diagnosis method	Relative risk of GDM compared with reference group					Potential study-level biases
Dishi et al. [18], USA	13	32.7	Omega Study, largely white pregnancy cohort	185 out of 3486 pregnancies, week 2628 50g glucose load test followed by 100g OGTT using ADA 2003 criteria [28]	1.1 (0.7, 1.7) (< = 11 years)	1.1 (0.8, 1.6) (12 years)	Ref. (13 years)	0.8 (0.5, 1.3) (14 years)	1.2 (0.8, 1.8) (> = 15 years)	Self-reported AAM, only included English speakers/readers
Chen et al. [13], U.S.A	12.6	34.1	Nurses’ Health Study II, largely white cohort of nurses	1404 out of 42109 pregnancies, self-reported GDM in any of their previous pregnancies	1.3 (1.1, 1.5) (<= 11 years)	1.0 (0.9, 1.2) (12 years)	Ref. (13 years)	0.9 (0.8, 1.0) (>= 14 years)		All participants were nurses, self-reported AAM, self-reported GDM
Li et al. [14], China	13.1	28.5	Healthy Baby Cohort Study, Chinese birth cohort	1015 out of 6900 pregnancies, week 2428 75g OGTT using IADPSG criteria [29]	1.4 (1.1, 1.7) (<= 11 years)	1.1 (0.9, 1.3) (12 years)	Ref. (13 years)	1.0 (0.8, 1.1) (14 years)	1.1 (0.9, 1.4) (>= 15 years)	Self-reported AAM
Schoenaker et al. [15], Australia	12.9	29	Australian Longitudinal Study on Women’s Health, largely white cohort of women	357 out of 4749 pregnancies, self-reported GDM in any of their previous pregnancies which had been diagnosed by a clinician using a 50 g glucose load test followed by a 75 g OGTT using Australasian Diabetes in Pregnancy Society 1998 criteria [30]	1.7 (1.2, 2.3) (<= 11 years)	1.2 (0.9, 1.5) (12 years)	Ref. (13 years)	0.9 (0.7, 1.3) (14 years)	1.1 (0.8, 1.6) (>= 15 years)	Self-reported AAM, self-reported GDM
Petry et al. [17], U.K	12.9	33.6	Cambridge Baby Growth Study, largely white birth cohort	87 out of 889 pregnancies, week 26–28 75g OGTT using IADPSG criteria [29]	3.7 (1.4, 9.7) (8–9 years)	1.1 (0.6, 1.9) (10–11 years)	Ref. (12–13 years)	1.0 (0.6, 1.6) (14–15 years)	2.4 (1.2, 2.9) (16–17 years)	Self-reported AAM, a smaller study than the others

Data are mean (with 95% CI in some cases) unless stated otherwise

*ADA* American Diabetes Association, *IADPSG* International Association of Diabetes in Pregnancy Study Group, *OGTT* oral glucose tolerance test, *Ref* signifies the reference group

### Sensitivity analysis

When adding the study by Shen et al. [16] to the other five studies included in the main meta-analysis, the evidence for a non-linear association between AAM and the future risk of developing GDM in pregnancy was strengthened slightly (overall *p* = 4.0 × 10^−9^; *p* for non-linearity = 1.0 × 10^−4^). The heterogeneity lessened: multivariate Cochran Q-test Q = 11.9 (10 degrees of freedom), *p* = 0.3, *I*^2^ = 16.2%. Again the largest predicted risk of GDM (relative to the risk associated with menarche at age 13 years) was associated with having a low AAM: 9 years 2.0 (1.6, 2.5), 10 years 1.6 (1.4, 2.0), 11 years 1.3 (1.2, 1.5), 12 years 1.1 (1.1, 1.1), 13 years was the reference, 14 years 1.0 (1.0, 1.0), 15 years 1.1 (0.9, 1.2), 16 years 1.2 (0.9, 1.4).

When excluding one study at a time from the main meta-analysis, although the degree of heterogeneity changed (from inconsequential to nearly moderate, the smallest heterogeneity occurring when the largest study was excluded), there was always a significant non-linear association between AAM and risk of developing GDM in pregnancy (Table 2). In each case, the predicted higher relative risk of developing GDM in pregnancy was associated with having an AAM of between 9 and 12. The highest risk was always associated with the earliest AAM.

**Table 2:** Sensitivity analysis of the main dose response meta-analysis performed by excluding each of the five studies included in the main analyses one at a time in turn

Included studies	Excluded studies	Overall *p* value	Non-linearity *p* value	Heterogeneity			Relative risk for GDM by age at menarche (years)							
				*Q* (6 d.o.f.)	*P*	*I* ^2^ (%)	9	10	11	12	13	14	15	16
Dishi, Chen, Li, Schoenaker [13–15, 18]	Petry	8.3 × 10^−^^9^	5.2 × 10^−^^4^	6.33	0.4	5.2	1.8 (1.5, 2.2)	1.5 (1.3, 1.7)	1.3 (1.2, 1.4)	1.1 (1.1, 1.1)	Ref	0.98 (0.94, 1.0)	1.00 (0.91, 1.1)	1.05 (0.88, 1.2)
Chen, Li, Petry, Schoenaker [13–15, 17]	Dishi	1.3 × 10^−^^6^	1.9 × 10^−^^3^	9.24	0.2	35.0	2.04 (1.56, 2.7)	1.65 (1.38, 2.0)	1.34 (1.22, 1.5)	1.12 (1.08, 1.2)	Ref	0.99 (0.94, 1.0)	1.05 (0.91, 1.2)	1.14 (0.89, 1.5)
Dishi, Li, Petry, Schoenaker [14, 15, 17, 18]	Chen	1.3 × 10^−^^6^	2.1 × 10^−^^5^	6.08	0.4	1.3	2.1 (1.56, 2.9)	1.7 (1.37, 2.1)	1.4 (1.20, 1.5)	1.1 (1.07, 1.2)	Ref	1.0 (0.97, 1.0)	1.1 (0.99, 1.2)	1.2 (1.03, 1.5)
Chen, Dishi, Petry, Schoenaker [13, 15, 17, 18]	Li	2.4 × 10^−^^5^	5.4 × 10^−^^3^	10.29	0.1	41.7	2.10 (1.53, 2.9)	1.69 (1.36, 2.1)	1.36 (1.21, 1.5)	1.12 (1.08, 1.2)	Ref	0.99 (0.94, 1.0)	1.05 (0.90, 1.2)	1.15 (0.87, 1.5)
Dishi, Chen, Li, Petry [13, 14, 17, 18]	Schoenaker	1.4 × 10^−^^8^	2.4 × 10^−^^4^	10.74	0.2	25.5	1.96 (1.58, 2.4)	1.61 (1.39, 1.9)	1.32 (1.22, 1.4)	1.11 (1.08, 1.1)	Ref	0.99 (0.95, 1.0)	1.05 (0.93, 1.2)	1.14 (0.93, 1.4)

Data are mean (95% CI)

*dof* degrees of freedom, *Ref*. signifies the reference group

### Evidence of publication bias

**Fig. 4:**
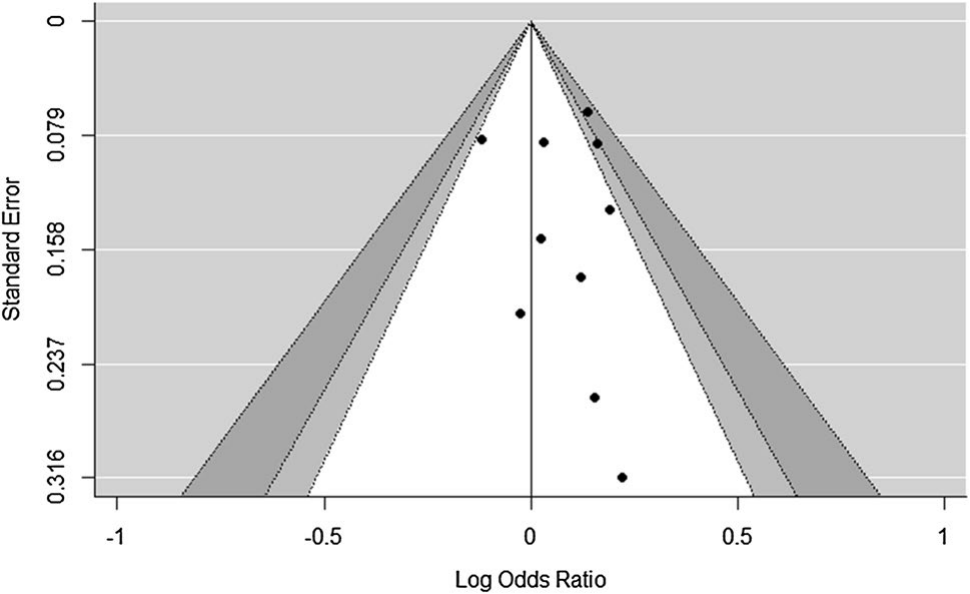
Funnel plot of the logarithmically transformed odds ratios and standard errors from pooled groups with AAM categories of less than 13 years and more than 13 years (with the categories containing an age at menarche of 13 years used as the reference). White is the 90% boundary, light grey is the 90–95% boundary and dark grey is the 95–99% boundary

**Fig. 5:**
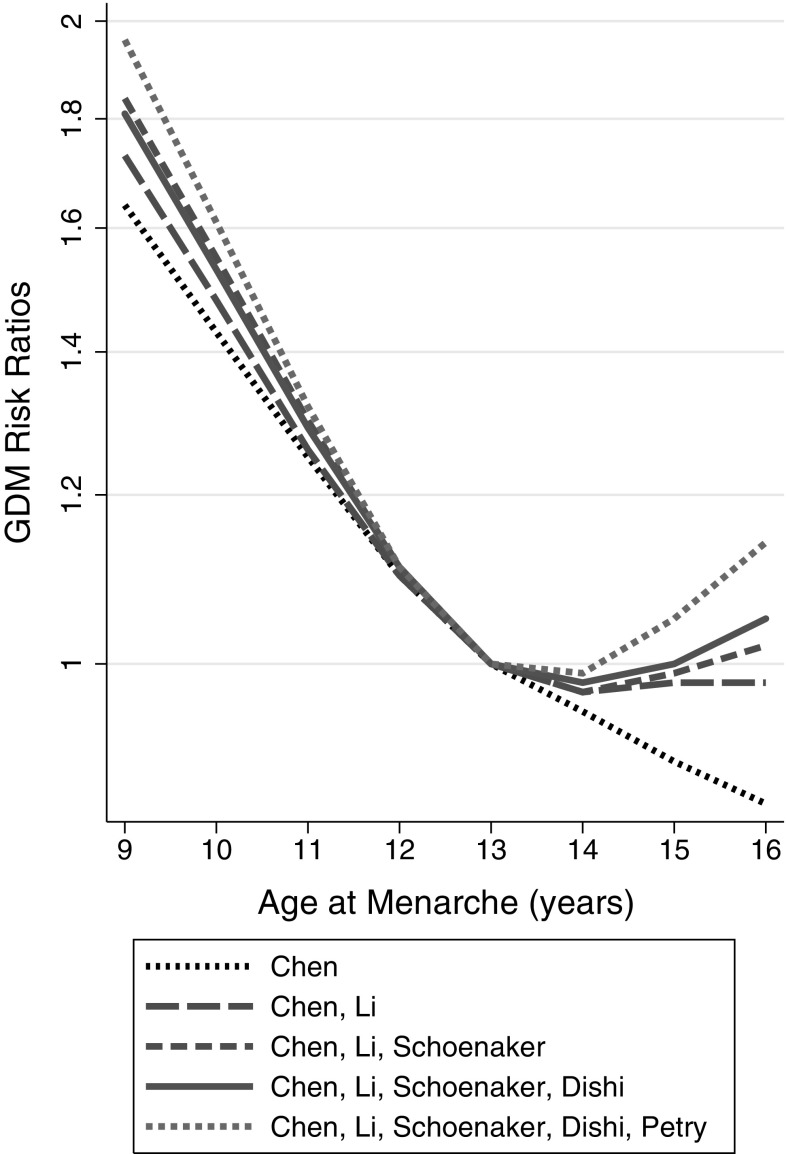
Pooled dose response associations between AAM and the risk of developing GDM in pregnancy relative to the risk associated with an AAM of 13 years. The different lines represent values from just using the largest study, then the two largest studies, then three, etc. until all the five studies were included in the analysis. The lines are labelled in the legend according to the first author of the manuscript describing each of the included studies. The associations with AAM were assessed in restricted cubic splines in multivariate random effects dose response models

A modified Egger’s regression test produced a significant result (*p* = 0.03, 95% confidence interval of the *p* value 0.01–0.14), although the test for heterogeneity was not significant (*Q* = 9.41 with 9 degrees of freedom, *p* = 0.4). The model estimated bias result on the odds ratio scale was 1.08 (1.01, 1.15). The funnel plot (Fig. 4) appeared asymmetric, with the majority of points located to the right of the reference line. Figure 5 illustrates the effect of including a smaller number of the studies on the risk ratios for GDM. There was a trend for an increasing relative risk for GDM with an AAM of 9–12 as smaller and smaller studies were added to the meta-analysis.

## Discussion

This meta-analysis of five prospective studies [13–15, 17, 18] has shown a significant, non-linear relationship between the AAM and the relative risk of developing GDM in pregnancy. Despite the fact that only five studies were included in the main analysis, the relationship appears to be reasonably strong. During the writing up of our study two other meta-analyses of the relationship between AAM and future risk of GDM were published [31, 32]. There are a number of differences between these published meta-analyses and the present meta-analysis, however, including: the inclusion of one study [16] in the main analysis of the previously published meta-analyses that we only incorporated into our study’s sensitivity analyses and not the main analyses due to the two published meta-analyses having slightly different inclusion criteria [31, 32], these previously published meta-analyses did not include data from our own recently published study [17] and the form of meta-analysis used in both analyses was not designed for dose response curves, so the comparison used was effectively between GDM risk in women with “early” and “not-early” AAM rather than taking a specific age as a reference age. Despite these differences, the relative risk or odds ratios for GDM in women with an early AAM relative to that of reference groups were remarkably similar between the three studies, the slightly lower values in [31, 32] possibly relating to those studies including the AAM categories that contributed to the non-linear relationship observed in the present analysis in their reference groups.

As far as a small meta-analysis such as this present analysis can be interpreted, there appears to be the possibility of publication bias having affected the relative risk. Egger’s test, which would have had low statistical power for testing funnel plot heterogeneity even if it was based on simple case–control studies rather than dose response curves [33], had to be adapted as it was not designed for data derived from dose response curves. We did this by pooling some of the AAM categories. Although the test may therefore have been run sub-optimally, it still gave a significant result. This result was consistent with the apparent asymmetry in the funnel plot (Fig. 4). It therefore seems fair to assume that there might have been an excess of studies with positive associations that have been published to date. Techniques such as Duval and Tweedie’s trim and fill [34] that are used in other meta-analyses in an attempt to overcome potential publication bias, are unsuitable for data derived from dose response curves. As an alternative which was suitable for use with data such as ours, Borenstein et al. [27] suggested reanalysing just the largest study, then adding the next largest study and reanalysing and continuing to do this whilst gradually adding successively smaller studies until all the studies are included. This method makes the assumption that larger studies are more likely to get published whether or not they produce significant associations (whereas smaller studies may only get studied if they show significant associations), and therefore the publication of larger studies is less dependent on bias. We demonstrated a positive increase in maximal relative risks for GDM as studies of decreasing size were added to the analysis (Fig. 5), adding the smallest study [17] causing the biggest increase in relative risks. All these meta-analyses showed an association with GDM, it was just the relative risk that changed. The true maximal relative risk for GDM may therefore be closer to 1.6 (the lower limit of the 95% confidence interval) than 2 for an AAM of 9 years, but the overall interpretation of this association is the same. The impact of any publication bias is therefore probably modest at best [27].

Not surprisingly from the fact that only five studies were included in the meta-analysis there was no statistically significant heterogeneity. Indeed the relevant I^2^ value of 25.5% suggests relatively low heterogeneity overall. This is despite the five studies used having some ethnic differences in their study populations and different cut-off circulating glucose concentrations used to define what constitutes GDM, both of which could have been associated with factors that contributed to increasing the heterogeneity. Interestingly when the initially excluded sixth study [16] was included in the meta-analysis for the sensitivity analysis the heterogeneity actually dropped. The results from the other sensitivity analyses, performed by excluding each one of the five studies from the meta-analysis in turn, also showed that despite the variation in the heterogeneity caused by each of the studies the material relationship between AAM and risk of developing of GDM in pregnancy did not change.

Although the relationship between AAM and the development of GDM in pregnancy that we found is non-linear, the meta-analysis has clarified the fact that in the studies published so far all the modelled significant increased risk for GDM is associated with earlier AAM. Early AAM tends to be associated with early closure of the epiphyseal plates and a relatively shorter stature in adult life [35]. It is subsequently associated with increased weight gain (relative to stature) and obesity [36], the strongest risk factor for GDM. Hence there is a link between early AAM and future GDM risk in pregnancy. In our original study [17] we also found a link between AAM and insulin resistance in pregnancy, and adjusting for insulin resistance completely attenuated the association between AAM and GDM. These results suggest that insulin resistance may be the primary factor underpinning the association between early AAM and increased GDM risk. Some studies have shown this association to be a linear inverse relationship [13], whereas others have shown more of a curvilinear relationship [16, 17] with additional increased relative risk, albeit smaller, for GDM being associated with older AAM. It could be that the bulk of the heterogeneity in the meta-analysis is at this end of the dose response curve, given that it dropped to an I^2^ of only 1.3% when the largest study was excluded, that study being one showing a linear rather than curvilinear relationship between AAM and GDM risk [13]. Although our meta-analysis showed a non-linear relationship, at present there is not enough evidence to suggest that there is a consistent association between older AAM and a raised future risk of GDM in pregnancy.

The strengths of this study include the fact that all the studies contributing to the meta-analysis were conducted prospectively in terms of GDM development and so should have potentially reduced recall and selection biases in comparison to if the studies were conducted retrospectively or in a case–control fashion. Another strength is the fact that the type of meta-analysis performed was specifically designed to test relationships in a dose response fashion [21], unlike the other meta-analyses published in this area [31, 32], and provide information about the shape of that relationship. Finally this meta-analysis covers more than 3,000 pregnancies affected by GDM, which is a very large number given its prevalence in the population as a whole, along with more than 55,000 unaffected pregnant women.

In addition to potential publication bias the limitations of this meta-analysis include the fact that it was based on data from only five different studies. There was only one published study presenting data relating AAM with future risk of GDM [16] that was not included in the final meta-analysis. That was primarily because this manuscript did not present unadjusted risk ratios or numbers of women with and without GDM from which they could be calculated. However it was included in the sensitivity analyses, where its presence did not materially change the interpretation of the relationship between AAM and risk of developing GDM in pregnancy suggesting that the results from the meta-analysis are sensitive. Another limitation is that no attempt was made to adjust the results for potential covariates. However the main potential covariate for GDM risk was maternal BMI, and it is debatable that this should have been controlled for given that in addition to the association with GDM risk, AAM also has a relationship with adult BMI [36]. This may actually reflect a single multifactorial process that includes AAM and starts even before birth [37] then includes effects on both adult BMI, and GDM risk in pregnancy as the woman ages. Another potential covariate that was not adjusted for was family history of diabetes. Only one of the studies showing an association between AAM and GDM risk presented data on, and a positive association between, family history of diabetes and GDM [16]. This was the study that was excluded from the meta-analysis for the reasons presented. One further study adjusted for it as a confounder in some of their statistical models [13], but did not present data on it. It remains a possibility, therefore, that a family history of diabetes may modify the relationship between AAM and GDM. A further limitation of this area of research, with the studies that have been published so far rather than with this meta-analysis, is the fact that the relationship between AAM and GDM risk in pregnancy has not been tested in sufficient numbers of women of non-white ethnicities. Future studies need to be performed in a wider range of ethnicities, particularly those that have the highest incidences of GDM.

In summary, this meta-analysis has shown that there is a significant relationship between AAM and the risk of GDM developing in subsequent pregnancy that is non-linear. Although there is evidence of possible publication bias in the results that have been published in this area to date, the effect of this appears to be an inflation of the relative risk from around 1.6 to closer to 2 rather than being anything that would alter the overall interpretation of the results. The risk appears to be around a younger AAM being associated with an increased risk of GDM. Further studies are required to clarify whether or not there is also an increased risk of GDM associated with having a relatively late AAM.

## Electronic supplementary material

Below is the link to the electronic supplementary material.

Supplementary material 1 (DOC 56 KB)
