# Supplementary material for: Age at menarche and the future risk of gestational diabetes: a systematic review and dose response meta-analysis
Source: Acta Diabetol. 2018 Aug 29;55(12):1209–19. doi: 10.1007/s00592-018-1214-z (PMC6244847; doi:10.1007/s00592-018-1214-z)
Supplement: Supplementary file 1 — Supplementary material 1 (DOC 56 KB) [file 592_2018_1214_MOESM1_ESM.doc]

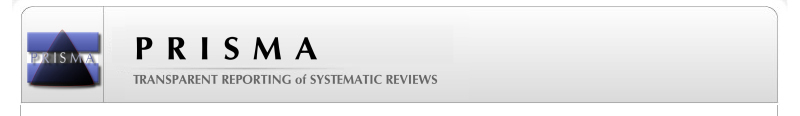
**PRISMA 2009 Flow Diagram**

**Screening**

**Included**

**Eligibility**

**Identification**

Records identified through database searching
(n = 125 )

Additional records identified through other sources
(n = 1 )

Records after duplicates removed
(n = 111 )

Records screened
(n = 111 )

Records excluded
(n = 100 )

Full-text articles assessed for eligibility
(n = 11 )

Full-text articles excluded, with reasons
(n = 6 )

Studies included in qualitative synthesis
(n = 5 )

Studies included in quantitative synthesis (meta-analysis)
(n = 5 )
